# High-Quality *de Novo* Genome Assembly of the *Dekkera bruxellensis* Yeast Using Nanopore MinION Sequencing

**DOI:** 10.1534/g3.117.300128

**Published:** 2017-08-09

**Authors:** Téo Fournier, Jean-Sébastien Gounot, Kelle Freel, Corinne Cruaud, Arnaud Lemainque, Jean-Marc Aury, Patrick Wincker, Joseph Schacherer, Anne Friedrich

**Affiliations:** * Génétique Moléculaire, Génomique, Microbiologie, Unité Mixte de Recherche 7156, University of Strasbourg, Centre National de la Recherche Scientifique, F-67000, France; † Commissariat à l’Energie Atomique, Institut de Biologie François-Jacob, Genoscope, BP5706, 91057 Evry, France

**Keywords:** genetic diversity, reference genome, population genomics, MinION sequencing, yeast, *Dekkera bruxellensis*, genome report

## Abstract

Genetic variation in natural populations represents the raw material for phenotypic diversity. Species-wide characterization of genetic variants is crucial to have a deeper insight into the genotype-phenotype relationship. With the advent of new sequencing strategies and more recently the release of long-read sequencing platforms, it is now possible to explore the genetic diversity of any nonmodel organisms, representing a fundamental resource for biological research. In the frame of population genomic surveys, a first step is to obtain the complete sequence and high-quality assembly of a reference genome. Here, we sequenced and assembled a reference genome of the nonconventional *Dekkera bruxellensis* yeast. While this species is a major cause of wine spoilage, it paradoxically contributes to the specific flavor profile of some Belgium beers. In addition, an extreme karyotype variability is observed across natural isolates, highlighting that *D. bruxellensis* genome is very dynamic. The whole genome of the *D. bruxellensis* UMY321 isolate was sequenced using a combination of Nanopore long-read and Illumina short-read sequencing data. We generated the most complete and contiguous *de novo* assembly of *D. bruxellensis* to date and obtained a first glimpse into the genomic variability within this species by comparing the sequences of several isolates. This genome sequence is therefore of high value for population genomic surveys and represents a reference to study genome dynamic in this yeast species.

Knowledge in biology has been greatly improved by exploring a large diversity of species as well as evolutionary contexts. No single species is representative of the evolution of either an entire phylum or a whole genus. Exploration of the genetic diversity of nonmodel species is essential to have a better insight into the variation of the population history, recombination, selection, mutation, and the genotype-phenotype relationship. In this context, the Saccharomycotina subphylum (budding yeasts), which includes the baker’s yeast *Saccharomyces cerevisiae*, represents an ideal group of nonmodel organisms for population genomic studies (Peter and Schacherer 2016).

Recent years have seen a burst of population genomic surveys focusing on various nonconventional yeasts associated with different objectives. This has a bearing on several aspects of evolutionary biology. Analysis of resequencing data of a large sample of isolates from the same species has been focused on yeast model organisms such as *S. cerevisiae* (Liti *et al.* 2009; Schacherer *et al.* 2009; Skelly *et al.* 2013; Bergström *et al.* 2014; Almeida *et al.* 2015; Strope *et al.* 2015; Gallone *et al.* 2016; Gonçalves *et al.* 2016; Zhu *et al.* 2016) and the fission yeast *Schizosaccharomyces pombe* (Fawcett *et al.* 2014; Jeffares *et al.* 2015), as well as on the nonmodel yeast species *Saccharomyces paradoxus* (Leducq *et al.* 2016), *Saccharomyces uvarum* (Almeida *et al.* 2014), *Candida albicans* (Ford *et al.* 2015; Hirakawa *et al.* 2015), and *Lachancea kluyveri* (Brion *et al.* 2015, 2016; Friedrich *et al.* 2015). Altogether, these data and analysis enhanced our knowledge about the evolutionary history of species (Almeida *et al.* 2014), the forces involved in genome evolution (Friedrich *et al.* 2015), and the genetic basis of the phenotypic diversity (Ford *et al.* 2015).

Among the Saccharomycotina, *Dekkera bruxellensis* is a yeast species associated with human fermentation processes that is well known as a major cause of wine spoilage, and also as an essential contributor to Belgium lambic and gueuze beer fermentation (Schifferdecker *et al.* 2014; Masneuf-Pomarede *et al.* 2015). In addition to its industrial properties, this species is of interest at the evolutionary level. Natural isolates show different ploidy levels (Borneman *et al.* 2014; Curtin and Pretorius 2014) and extensive chromosomal rearrangements, which were observed through electrophoretic karyotypes (Hellborg and Piškur 2009). These observations indicate a rapid evolution at the intraspecific level. Recent findings suggest that the ploidy level could be linked to the substrate of origin of the strain and related to adaptive processes linked to specific environments (Albertin *et al.* 2014). Consequently, a genome-wide polymorphism survey based on a representative set of *D. bruxellensis* individuals would be of interest. The exploration of single nucleotide polymorphisms (SNPs), small indels, as well as structural variants such as large indels, and inversions and translocations at the species level would help provide insight into the forces that shape genomic architecture and evolution. However, to conduct a population genomic survey, the availability of a high-quality reference sequence for the species at a completeness level to cover the majority of the genomic variation and a contiguity level to efficiently detect structural variants, is a prerequisite.

To date, population genomic studies have mostly been performed on species for which chromosomal-scale genome assemblies were available; however, this necessary high-quality assembly was unfortunately not yet available for the *D. bruxellensis* species. Here, we present the *de novo* sequence and high-quality genome assembly of the UMY321 *D. bruxellensis* isolate with a combination of long Oxford Nanopore and short Illumina reads. By aligning the short-read sequencing data from a total of eight sequenced natural isolates on the generated assembly, as well as other previously available assemblies (Curtin *et al.* 2012; Piškur *et al.* 2012; Borneman *et al.* 2014; Crauwels *et al.* 2014; Olsen *et al.* 2015), we tested the capacity of our assembly to be used as a reference assembly for future population genomic studies of this nonmodel species. The results showed that we generated the most complete and contiguous *de novo* assembly of *D. bruxellensis* necessary to explore the intraspecific genetic diversity of this unique and economically relevant species.

## Materials and Methods

### Yeast strains and DNA preparation

We selected three *D. bruxellensis* diploid isolates from various ecological and geographical origins (Table 1). The UMY321 isolate was chosen for the generation of a high-quality assembly and was therefore subjected to Oxford Nanopore and Illumina sequencing. The two other isolates, UMY315 and 133, were only subjected to Illumina sequencing for comparative analysis purposes.

**Table 1:** Description of the *D. bruxellensis* isolates used in this study

Strain	Ploidy	Ecological Origin	Geographical Origin	Reference
AWRI1499	3n	Wine	Australia	Curtin *et al.* (2012)
AWRI1608	3n	Wine	Australia	Borneman *et al.* (2014)
AWRI1613	2n	Wine	Australia	Borneman *et al.* (2014)
CBS11270	2n	Industrial ethanol	Sweden	Olsen *et al.* (2015)
CBS2499	2n	Wine	France	Piškur *et al.* (2012)
ST05_12_22	2n	Lambic beer	Belgium	Crauwels *et al.* (2014)
UMY315	2n	Must	Italy	This study
UMY321	2n	Red wine	Italy	This study
133	2n	Merlot wine	South Africa	This study

Yeast cell cultures were grown overnight at 30° in 20 ml of YPD medium to early stationary phase before cells were harvested by centrifugation. Total genomic DNA was than extracted using the QIAGEN Genomic-tip 100/G according to the manufacturer’s instructions.

### Flow cytometry

Samples were prepared for DNA content analysis using flow cytometry. Cells were grown in YPD medium at 30° to reach exponential phase. They were then pelleted and washed with 1 ml water. In order to fix the cell, the pellet was resuspended in 1 ml of 70% ethanol. After centrifugation, supernatant was discarded and cells were resuspended in 1 ml sodium citrate buffer (trisodium citrate 50 mM; pH 7.5). Cells were pelleted once more and resuspended in 1 ml sodium citrate buffer supplemented with 10 µl of RNase A (100 mg/ml) and incubated at 37° for 2 hr. Samples were then sonicated (Sonics Vibra-Cell VC750) for 20 sec with a 20% amplitude. After sonication, 1 ml sodium citrate buffer supplemented with 10 µl propidium iodide (1.6 mg/ml) and left in the dark at 4° for 12 hr. Once the cells were stained with propidium iodide, cell DNA content was assessed by measuring fluorescence intensity using flow cytometry (CyFlow Space; Partec).

### MinION library preparation and sequencing

We sheared 2 μg genomic DNA to ∼8000 bp with g-TUBE. After clean-up using 1× AMPure XP beads, Nanopore’s 8-kb two-dimensional (2D) sequencing libraries were prepared according to the SQK-MAP005-MinION gDNA Sequencing Kit protocol.

The sequencing mix was prepared with 8 μl of the DNA library, water, Fuel Mix, and Running buffer, according to the SQK-MAP005 protocol. The sequencing mix was added to the R7.3 flowcell for a 48 hr run. The flowcell was reloaded one time at 24 hr with an addition of 8 μl of the DNA library.

### Illumina sequencing

Genomic Illumina sequencing libraries were prepared with a mean insert size of 280 bp and were subjected to paired-end sequencing (2 × 100 bp) on Illumina HiSeq2000 sequencers.

### De novo genome assembly

Various sets of the longest MinION 2D reads, which refer to various theoretical genome coverage (10×, 15×, 20×, and all the 2D reads, *i.e.* ∼25×) taking 15 Mb as genome size estimate (Supplemental Material, Table S1 in File S1), were subjected to four assemblers: ABruijn (v0.3b) (Lin *et al.* 2016), Canu (v1.1) (Berlin *et al.* 2015), miniasm (v0.2-r137-dirty) (Li 2016), and SMARTdenovo (https://github.com/ruanjue/smartdenovo). ABruijn and miniasm were run with default parameters, while “genomeSize=13 m, minReadLength=2500, mhapSensitivity=high, corMhapSensitivity=high, and corOutCoverage=500” was set for Canu and “-c 1 -k 14 -J 2500 -e zmo” for SMARTdenovo. After the assembly step, we polished each set of contigs with Pilon (v1.18) (Walker *et al.* 2014), using ∼100× of Illumina 2 × 100 bp paired-end reads. SSPACE-LongRead (v1.1) (Boetzer and Pirovano 2014) was finally used to scaffold the selected assembly using long-reads information.

### Assembly completeness evaluation

The completeness of our assembly was evaluated firstly, through the proportion of unmapped short reads (see *Short-read mapping*) determined with Samtools (v0.1.19) (Li *et al.* 2009) using the option “view -f 4 -c”; and secondly, through the proportion of ultraconserved core eukaryotic genes recovered by CEGMA (v2.5) (Parra *et al.* 2007), with default parameters.

### Whole genome comparison

Whole genome comparisons were performed with MUMmer (v3.0) (Kurtz *et al.* 2004). nucmer was used to align the sequences (with –maxmatch option). The alignments coordinates were extracted to determine the proportion of non-N residues of each assembly that were covered. The delta files were filtered for alignments <5 kb and plots were generated with mummerplot.

### Short-read mapping

Reads were mapped with BWA (v0.7.4) (Li and Durbin 2009) and unmapped reads were estimated with Samtools (v0.1.19) (Li *et al.* 2009). GATK (v3.3) (McKenna *et al.* 2010) was used for local realignment of the reads around indels, SNPs calling, and to add allele balance information in the vcf file.

### Data availability

All sequencing data generated in this study, as well as the UMY321 reference assembly (in FASTA format), have been deposited in the European Nucleotide Archive under the accession number PRJEB21262.

## Results and Discussion

Three *D. bruxellensis* isolates (UMY321, UMY315, and 133) were sequenced in this study (Table 1). These strains were determined to be diploid based on flow cytometry analysis and were all isolated from wine or grape must in Italy or South Africa. The genome of the UMY321 isolate was sequenced using a combination of Nanopore long-read and Illumina short-read sequencing data to obtain a high-quality assembly. By contrast, the UM315 and 133 isolates were only sequenced using a short-read strategy. In addition, these genomes were compared to previously genome sequences of six other *D. bruxellensis* isolates (Table 1) (Curtin *et al.* 2012; Piškur *et al.* 2012; Borneman *et al.* 2014; Crauwels *et al.* 2014; Olsen *et al.* 2015).

### De novo genome assembly construction and comparison

For the UMY321 isolate, a total of three MinION Mk1 runs were performed with the R7.3 chemistry using 2D library types with 8 kb mean fragmentation size. A total of 115,559 reads representing a cumulative size of 1.15 Gb were generated, among which 41,686 2D reads showed an average quality greater than nine (2D pass reads). We focused on these 2D pass reads representing a total of 376.8 Mb, with the longest read being 70,058 bp (mean = 9033 bp and median = 8676 bp) (Figure S1). Four subsets of our 2D pass reads (10×, 15×, and 20× of the longest 2D pass reads, and all of them, *i.e.* ∼25×) (Table S1 in File S1) were submitted to four assemblers: ABruijn (Lin *et al.* 2016), Canu (Berlin *et al.* 2015), miniasm (Li 2016), and SMARTdenovo (https://github.com/ruanjue/smartdenovo). As the MinION sequencing technology is known to be associated with high error rates (∼10% for 2D pass reads) (Jain *et al.* 2016), we polished the assemblies with Pilon (Walker *et al.* 2014) using 100× of Illumina paired-end reads. The lengths of the constructed assemblies were all in the same order of magnitude and ranged from 11.7 to 13.7 Mb (Table S2 in File S1).

**Figure 1:**
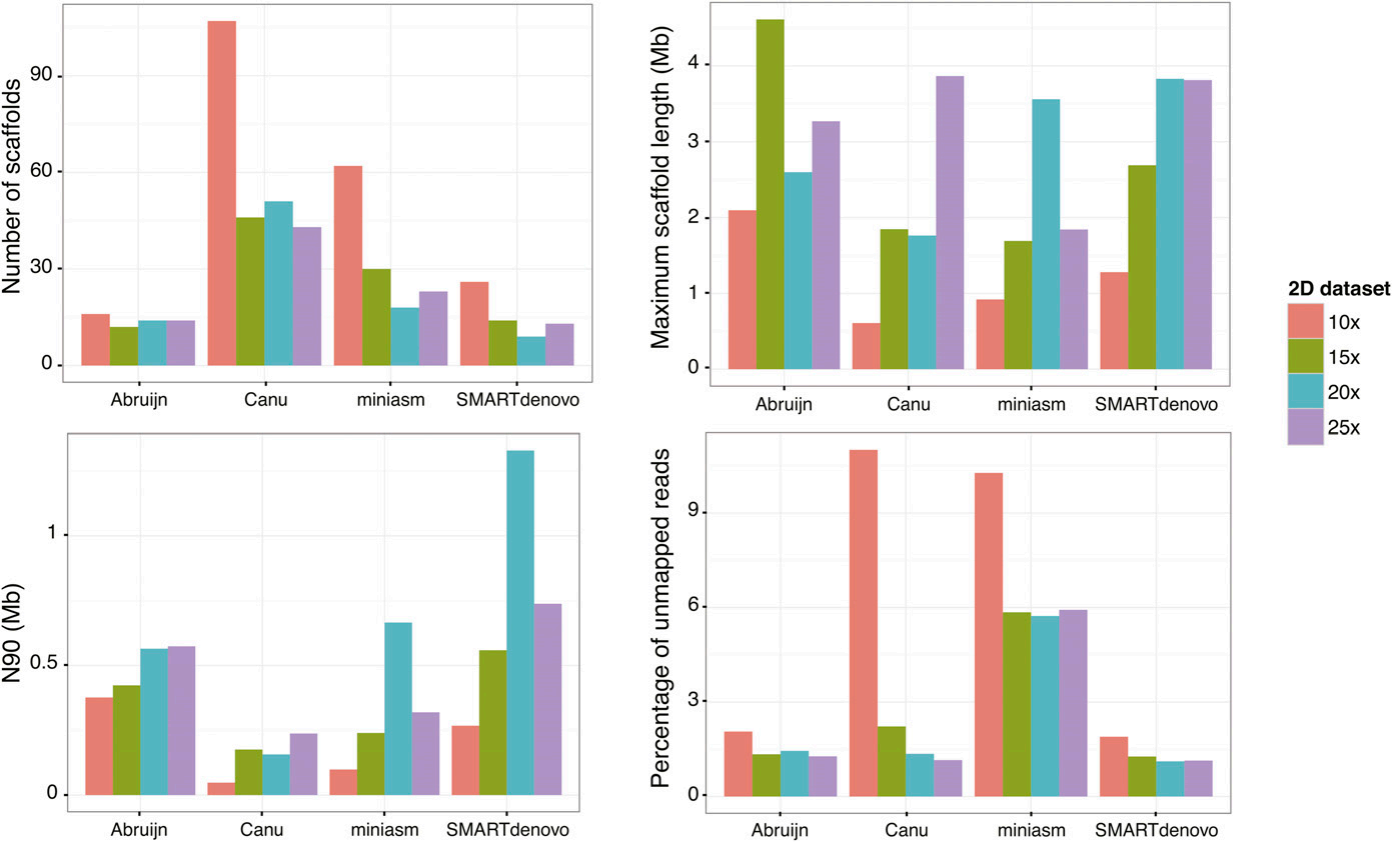
Metrics related to the constructed assemblies, per assembler and dataset.

Using these various datasets and assemblies, the objective was to define the best assembler and the minimal coverage needed. Hence, we computed the standard contiguity metrics for all assemblies to evaluate their quality, which is related to both the assembler and the dataset (Figure 1 and Table S2 in File S1). First, we observed that, considering the results by assembler, the number of scaffolds obtained with the 10× dataset is much higher compared to the other datasets, which suggests that a 10× coverage of MinION reads is too low to obtain a good quality assembly. By assembler, the results obtained for the higher coverages are comparable. Using Canu, the number of scaffolds is much higher and N90 as well as N50 are much lower, producing the less connected assemblies (Figure 1 and Table S2 in File S1). The contiguity metrics associated with the assemblies constructed with SMARTdenovo, ABruijn, and miniasm were closely related, and it seemed difficult to select a single best assembly on the sole basis of these measurements, especially since good contiguity metrics are not necessarily associated with assembly completeness.

Considering these results, we decided to map the Illumina paired-end reads back on the generated assemblies with BWA (Li and Durbin 2009). Among all the assemblies, the proportion of unmapped reads ranged from 1.12 to 11% (Figure 1 and Table S2 in File S1). Surprisingly, the assemblies constructed with miniasm were less complete, as >5% of the reads did not map back, compared to <1.5% for the ABruijn and SMARTdenovo assemblies.

By comparing standard metrics and the proportion of unmapped reads, the most accurate assembly was obtained with the 20× 2D reads dataset combined with the SMARTdenovo assembler. This assembly is composed of nine scaffolds, *i.e.*, very close to the estimated number of chromosomes, which appears to vary between four and nine among different strains of this species (Hellborg and Piškur 2009), for a complete assembly size of 12.97 Mb. This was then submitted to SSPACE-longreads, which reduced the number of scaffolds to eight after grouping the two smallest ones, based on our long-read information, and a further Pilon run. The final assembly contains eight scaffolds and shows a cumulative size of 12,965,163 bp (Table 2). We also evaluated the completeness of our assembly at the gene content level by running CEGMA (Parra *et al.* 2007): 245 out of the 248 most extremely conserved genes in eukaryotes were detected in our assembly, through 242 complete and three partial alignments. Altogether, these results reveal a high level of completeness of our assembly.

**Table 2:** Metrics associated with the *D. bruxellensis* publicly available assemblies

Strain	No. of Scaffolds	Assembly Size (Mb)	Maximum Scaffold Size	N50	N90	No. of Undetermined Residues
AWRI1499 (Curtin *et al.* 2012)	324	12.7	170,307	65,420	22,583	57
CBS11270 (Olsen *et al.* 2015)	15	17.3	4,993,495	3,706,654	944,992	2,497,785
CBS2499 (Piškur *et al.* 2012)	84	13.4	2,877,306	1,792,735	190,560	586,105
ST05_12_22 (Crauwels *et al.* 2014)	85	13.1	1,439,423	732,210	177,142	218,317
UMY321 (this study)	8	13	3,829,289	1,917,156	1,329,398	2708

### Comparison with available assemblies of D. bruxellensis

To date, several assemblies of the *D. bruxellensis* species have already been released (Curtin *et al.* 2012; Piškur *et al.* 2012; Borneman *et al.* 2014; Crauwels *et al.* 2014; Olsen *et al.* 2015). These assemblies are related to isolates from different ecological and geographical origins (Table 1). They were mostly constructed by combining several sequencing methods, such as 454, PacBio, and Illumina, as well as optical mapping in the most recently published assembly (Olsen *et al.* 2015).

The assemblies have very variable metrics associated with each of them (Table 2). In terms of contiguity, our assembly and the assembly generated for the CBS11270 isolate are close, and reach a chromosome-scale resolution. However, the CBS11270 assembly is much larger than the others (17.3 Mb *vs.* 12.7–13.4 Mb), although it does also contain ∼2.5 Mb of undetermined (N) residues.

By comparing the assembly metrics, we determined that our assembly is closer to that for CBS11270, which was generated by combining PacBio and Illumina sequencing methods as well as optical mapping, and much better than the other three available for comparison, which were much more fragmented and comprised at least 84 scaffolds.

**Figure 2:**
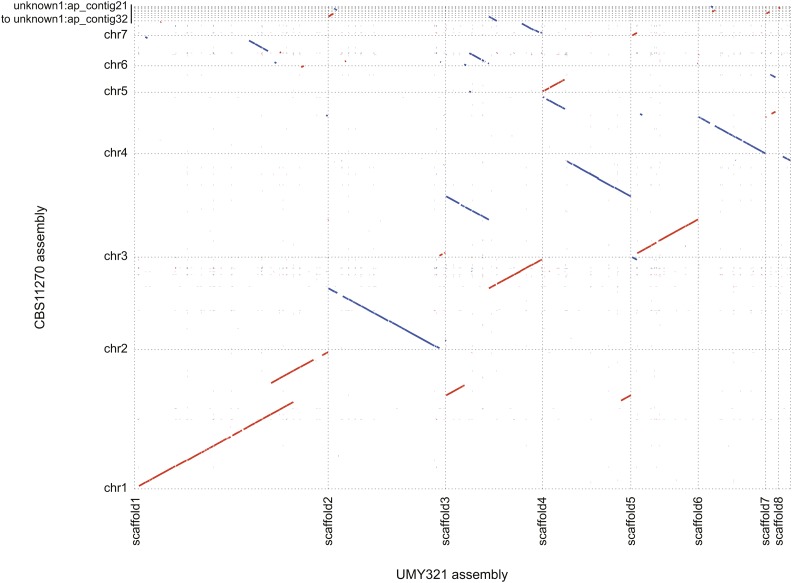
Comparison of the CBS11270 and UMY321 assemblies. The alignments and the plot were generated with the MUMmer software suite. Red lines: sequences aligning in the same direction. Blue lines: sequences aligning in the opposite direction.

A MUMmer comparison of our UMY321 assembly to that of CBS11270 indicates that 91 and 99.6% of the assemblies aligned, respectively, with one another and revealed that the scaffolds are mostly collinear (Figure 2). However, some large repetitive regions can be observed in the CBS11270 assembly, *e.g.*, on chromosome 1, between chromosomes 1 and 6, and between chromosomes 4 and 5 (Figure 2 and Figure S2) that are absent in our assembly, and could explain the size differences between the assemblies (17.3 Mb *vs.* 12.97 Mb). Moreover, some synteny breaks can be observed, at the level of scaffolds, specifically between three and four. All the inconsistencies between the assemblies could be related either to structural rearrangements between the isolates or to assembly errors, and would require further investigations to reach a conclusion as to their most likely source.

### Suitability of our assembly for population genomics studies

As previously mentioned, to function as a valuable resource for conducting population genomics studies, a reference genome should combine high contiguity (for the detection of structural variants) and completeness (for the efficient detection of SNPs and small indels). At the contiguity level, our assembly is close from a chromosomal-scale resolution, which suggests that it would be highly suitable for gross structural rearrangement detection (translocations, inversions, and long insertions/deletions).

**Figure 3:**
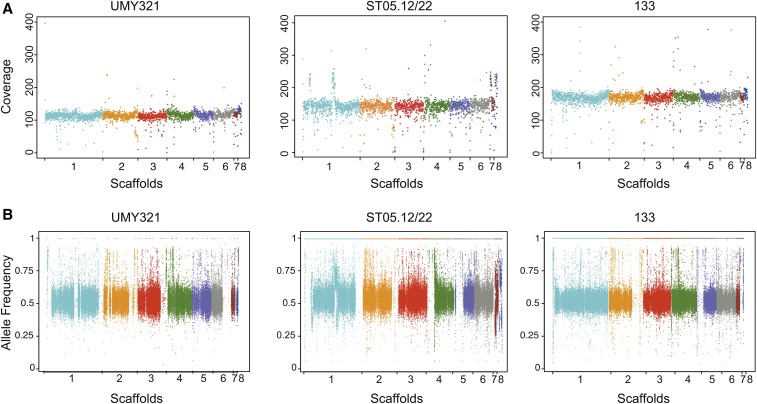
Mapping of the Illumina reads *vs.* the UMY321 reference assembly. (A) Illumina reads coverage along the reference genome. (B) Frequency of the reference allele at heterozygous sites along the genome. (Each color corresponds to a scaffold).

To test our assembly for the detection of polymorphism along the genome, we further investigated the mapping of the Illumina reads. As previously mentioned, 98.89% of the UMY321 Illumina reads mapped on our assembly. The read coverage was homogeneous along the scaffolds (Figure 3A), which suggests that the strain is devoid of aneuploidy and segmental duplication, and confirms the lack of large repetitive regions within our assembly.

A total of 83,006 SNPs were detected with GATK (McKenna *et al.* 2010), among which 374 were homozygous and 82,632 were heterozygous (Table S3 in File S1). The 374 homozygous SNPs could be considered as false positives. Although not completely negligible, this number is very low and could be related to the high error rate of the MinION technology, which is not completely compensated by using Illumina short reads (Istace *et al.* 2017).

The UMY321 isolate that we sequenced is diploid, and the detection of these 82,632 heterozygous SNPs revealed that the two genomic copies are not identical and have a high heterozygosity level. These heterozygous positions are mostly evenly distributed all along the genome, with several regions showing loss of heterozygosity (LOH) on scaffolds 1, 2, 3, and 6 (Figure 3B).

**Figure 4:**
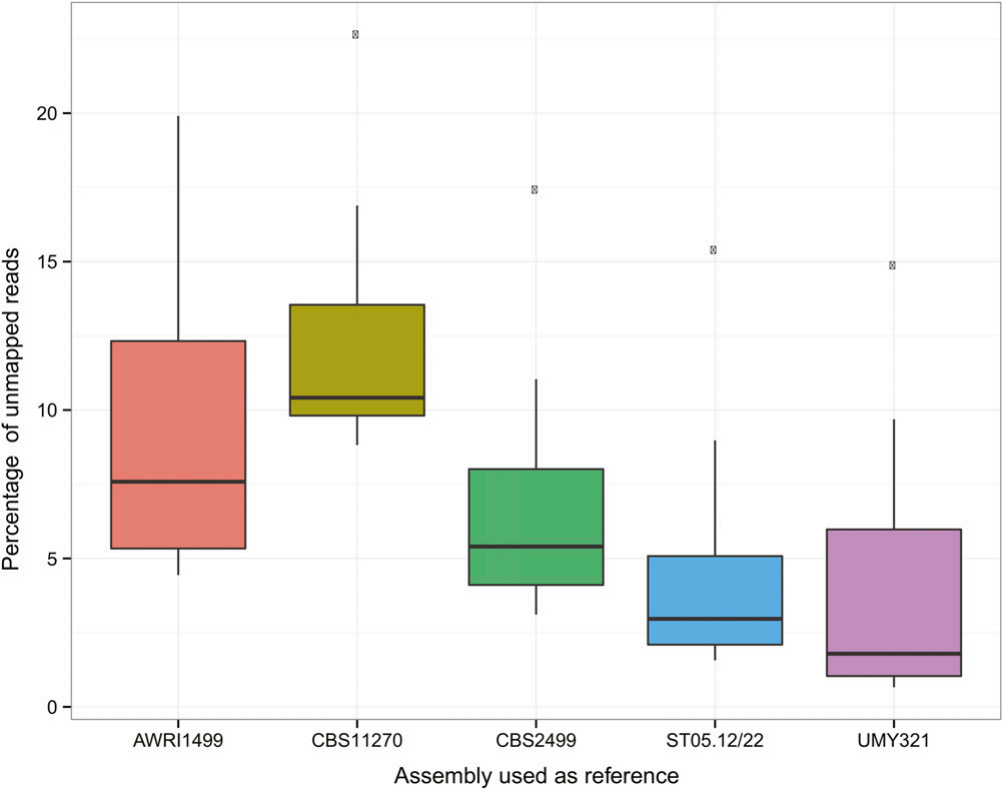
Illumina unmapped reads per assembly. Boxplot of the percentage of unmapped Illumina reads, according to the assembly used for the mapping.

Altogether, these results confirmed that our assembly performs well when mapping the reads that were used for its construction. However, to determine if an assembly is relevant in the context of population genomic studies, we also analyzed its performance when mapping reads from other isolates. To survey polymorphisms within a species, resequencing projects rely mainly on Illumina sequencing technology, therefore we mapped the short reads related to this species that were publically available as well as from two isolates we sequenced in the context of this project (Table S4 in File S1) against our assembly and reported the proportion of unmapped reads. We also aligned these reads against the publicly available assemblies to perform a comparative analysis (Table 3). As expected, the UMY321 Illumina paired-end reads mapped better on our assembly with only 1.11% of unmapped reads. More surprisingly, short reads generated in the context of the other projects also mapped better on our assembly compared to their related assemblies, and more generally compared to all other assemblies (Figure 4). It is also worth noting that all the reads, including those related to the CBS11270 isolate, mapped less efficiently to the CBS11270 assembly compared to all other assemblies, which suggests that although this assembly is highly contiguous and much larger than the others available, it is less complete.

**Table 3:** Proportion of *D. bruxellensis* unmapped Illumina reads on the available assemblies

		Assemblies				
		UMY321	CBS11270	CBS2499	ST05.12/22	AWRI1499
Illumina paired-end reads	UMY321	1.11	9.95	4.13	2.12	5.29
	CBS11270	4.74	12.43	5.88	3.48	9.49
	CBS2499	1.68	9.4	4.92	2.45	5.68
	ST05.12/22	1.9	10.83	7	3.78	11.97
	UMY315	0.66	10.00	4.04	2.02	5.35
	133	0.82	8.82	3.11	1.57	4.44
	AWRI1608	14.87	22.65	17.42	15.39	19.91
	AWRI1613	9.69	16.89	11.04	8.98	13.38

### Insight into the intraspecific genetic variability

Finally, we took advantage of the availability of Illumina reads related to different isolates in order to obtain a first glimpse into the genomic variability within this species, using our UMY321 assembly as a reference. The read coverage along the reference sequence was mostly homogeneous for all isolates, and only few deviations were observed, limited to small genomic regions, which are characteristic of segmental duplications, in the ST05.12/22 isolate (Figure 3A). This suggests that the structural variants within this species are mostly balanced. It can also be noticed that the coverage plot obtained from the CBS11270 Illumina reads did not show twofold deviations on scaffolds 1, 3, or 4 (data not shown), as expected from the comparison of the CBS11270 and UMY321 assemblies (Figure 2 and Figure S2), suggesting that the repetitive regions highlighted in the CBS11270 assembly are most probably related to assembly errors.

Among the eight studied isolates, one is triploid (AWRI1608) and all the others are diploid (Table 1). A total of 1,268,172 SNPs were detected across these eight isolates, among which 82% are heterozygous (Table S3 in File S1). These SNPs are distributed over 500,707 polymorphic positions, with a majority present as singletons (68.8% of the polymorphic sites). However, a significant proportion of this variability is related to the triploid strain AWRI1608. Indeed, when this strain was not included in the analysis, 829,313 SNPs were detected over 188,717 polymorphic positions with only 50,702 singletons (27%). This is in agreement with the proposition that AWRI1608 consists of a slightly heterozygous diploid set of chromosomes with an additional full set of more distantly related chromosomes (Borneman *et al.* 2014). The phylogenetic relationships between this small sample of isolates based on the whole set of polymorphic positions also reflect the high divergence of this triploid isolate (Figure S3A). Ploidy levels across the genomes were also confirmed by taking advantage of allele frequency at heterozygous positions, which was ∼0.5 for diploid isolates and 0.33/0.66 for the AWRI1608 genome (Figure S3B). These heterozygous positions are evenly distributed along the genome; however, LOH regions were detected in all the diploid isolates (Figure 3B).

### Conclusions

*D. bruxellensis* is a yeast species of great importance in fermented beverage industries, largely thought of as a contaminant organism (Schifferdecker *et al.* 2014; Masneuf-Pomarede *et al.* 2015). This species is also an interesting model to study genome evolution and dynamics as it is characterized by a large genomic plasticity. For these reasons, we sought to generate a high-quality genome assembly and ultimately obtain a suitable reference genome for population genomics. Our analyses show that the *D. bruxellensis* assembly that we generated with a combination of moderate coverage (20×) MinION long-reads in addition to a higher coverage (100×) of Illumina reads utilized for sequence polishing purposes, is highly valuable for population genomic studies and outperforms previously available sequences. Preliminary comparison among a small set of nine isolates already highlights the presence of large regions of LOH, which appears to be key factor in the genome evolution and adaptation of a large number of yeast species (Magwene *et al.* 2011; Ford *et al.* 2015; Smukowski Heil *et al.* 2017). To obtain a species-wide view of the genetic variability of *D. bruxellensis*, many more isolates should be surveyed using both short-read as well as long-read sequencing techniques, which will allow for the exploration of the structural variant landscape.

## Supplementary Material

Supplemental material is available online at www.g3journal.org/lookup/suppl/doi:10.1534/g3.117.300128/-/DC1.
